# SSZ‐27: A Small‐Pore Zeolite with Large Heart‐Shaped Cavities Determined by Using Multi‐crystal Electron Diffraction

**DOI:** 10.1002/anie.201905049

**Published:** 2019-08-21

**Authors:** Stef Smeets, Stacey I. Zones, Dan Xie, Lukáš Palatinus, Jesus Pascual, Son‐Jong Hwang, Joel E. Schmidt, Lynne B. McCusker

**Affiliations:** ^1^ Department of Materials and Environmental Chemistry Stockholm University 10691 Stockholm Sweden; ^2^ Kavli Institute of Nanoscience Delft University of Technology 2629 HZ Delft The Netherlands; ^3^ Chevron Energy Technology Company Richmond CA 94802 USA; ^4^ Institute of Physics of the Czech Academy of Sciences Na Slovance 2 Prague Czech Republic; ^5^ Division of Chemistry and Chemical Engineering California Institute of Technology Pasadena CA 91125 USA; ^6^ Department of Materials ETH Zurich Vladimir-Prelog-Weg 5 8093 Zurich Switzerland; ^7^ Department of Chemical Engineering University of California, Santa Barbara CA 93106 USA

**Keywords:** zeolites, electron diffraction, SSZ-27, structure determination, structure-directing agents (SDAs)

## Abstract

The high‐silica zeolite SSZ‐27 was synthesized using one of the isomers of the organic structure‐directing agent that is known to produce the large‐pore zeolite SSZ‐26 (**CON**). The structure of the as‐synthesized form was solved using multi‐crystal electron diffraction data. Data were collected on eighteen crystals, and to obtain a high‐quality and complete data set for structure refinement, hierarchical cluster analysis was employed to select the data sets most suitable for merging. The framework structure of SSZ‐27 can be described as a combination of two types of cavities, one of which is shaped like a heart. The cavities are connected through shared 8‐ring windows to create straight channels that are linked together in pairs to form a one‐dimensional channel system. Once the framework structure was known, molecular modelling was used to find the best fitting isomer, and this, in turn, was isolated to improve the synthesis conditions for SSZ‐27.

## Introduction

A number of novel high‐silica zeolite structures have been found through exploratory synthesis trials that combine an organic guest molecule, usually a quaternary ammonium compound, with a variety of synthetic conditions. We have recently described how the inorganic conditions chosen in the hydrothermal reaction can bias the crystalline product in terms of the sub‐units likely to occur in the zeolite structure.^1^ For example, syntheses can be tuned or modified to produce zeolites with relatively moderate SiO_2_/Al_2_O_3_ ratios (SAR) and small pores running multi‐dimensionally between cages (where the organic guest is found in the as‐synthesized zeolite). This is particularly relevant in the search for materials with high performance in DeNOx reactions in the SCR (selective catalytic reduction) components in diesel emission systems,^2^ in which there is high interest in novel small‐pore zeolites. High hydroxide levels and the presence of one of a number of alicyclic ring derivatives produce zeolites yielding an optimum DeNOx catalyst once the counter ions have been exchanged with copper or iron cations. In the various zeolite framework types discovered over the last few decades,^3^ there is a wide range of channel and cage architectures to choose from. A description of various unique zeolitic features with examples are given in Table S1 (Supporting Information).

**Figure 1:**
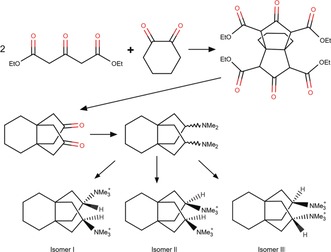
Synthesis scheme to make the diquaternary ammonium isomers used in SSZ‐26 (**CON**) and their labelling. Isomer III is used in the synthesis of SSZ‐27.

Herein we explore how the synthesis of a diquaternary organic structure‐directing agent (SDA) results in different isomeric versions of the SDA, and how a small difference can lead to the formation of a different zeolite framework. Figure 1 shows the synthesis path that produces the isomers and their labelling from an earlier work.^4^ Initially, we focused on the production of zeolite SSZ‐26 (**CON**) and showed that it could be made from two well‐described isomers (isomers I and III).^4^ The concept for developing these isomers was to break the fit of an SDA into either 1) a cage accessed by smaller portals, or 2) a single large pore. A past study, juxtaposing the size of the SDA and the SAR conditions, shows a breakdown into 5 types of zeolite products.^5^ At the time, we succeeded in creating a material with intersecting pores. In fact, this was the first discovery of a zeolite (SSZ‐26) with both large‐ and medium‐sized pores.^6^ During the course of the synthesis explorations for SSZ‐26, we occasionally encountered a different product. Early microporosity studies on the impurity phase, which we termed SSZ‐27, indicated that it had only small pores and selective hydrocarbon uptake. At the time, these materials were not of much developmental interest for catalysis or for separations, so further characterization of SSZ‐27 was not pursued. However, interest in small pore zeolites has recently taken a dramatic upward turn. We therefore revisited the synthesis of SSZ‐27, and show here that the conformation of one of the isolated isomers of the diquaternary SDA (isomer III) is such that it fits particularly well into one of the cavities of SSZ‐27. Here we present the reaction conditions and modelling results that led to the synthesis of SSZ‐27 and to an understanding of how it forms, and its structure determination from electron diffraction data.

## Results and Discussion

### Synthesis

Our point of departure was the synthesis of the diquaternary SDA, shown in Figure 1. The concept was to build on the established organic synthesis chemistry of Ginsberg and others^7^, ^8^ to build fused molecules called propellanes. In this way we could make derivatives that might promote the construction of channels in the zeolite framework that would extend in more than one direction. We could convert the resulting dione to a di‐tertiary amine in one step using the Leuckart reaction.^9^ These diamines can then be quaternized using methyl iodide. Note that in the formation of the diamine, the isomeric possibilities (there are 3 at this step) are fixed. Individual isomers were recovered by selective recrystallization methods and their structures determined using single‐crystal X‐ray diffraction data.^4^

**Figure 2:**
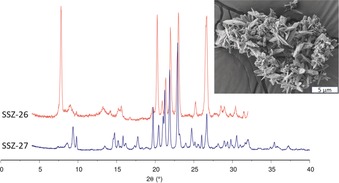
XRPD pattern for SSZ‐26 (**CON**) and SSZ‐27, both as‐synthesized. The inset shows an SEM image of as‐synthesized SSZ‐27. Note that the SSZ‐27 sample shown may contain an SSZ‐26 impurity (see text).

For the zeolite synthesis, we used a set of inorganic component ratios consistent with earlier work on high silica zeolites like ZSM‐5 (**MFI**), where a reduced hydroxide to silica ratio was needed to create structures rich in 5‐ring sub‐units. An important finding proved to be the need to reduce the alkali content while retaining the amount of hydroxide, and this was achieved by replacing the alkali cations with the quaternary ammonium centres. This change reduces the intrusion of high‐silica zeolite competitors such as Mordenite and Magadiite, which require sodium in the synthesis.^10^ As we were exploring the synthesis conditions for SSZ‐26, we occasionally encountered SSZ‐27 as a by‐product. SSZ‐27 formed well‐defined homogenous crystals (Figure 2). A comparison of the X‐ray powder diffraction patterns for SSZ‐26 and SSZ‐27 is shown in Figure 2. Experimental details of the SDA and zeolite synthesis can be found in the Supporting Information.

### Structure Determination using Electron Diffraction Data

**Figure 3:**
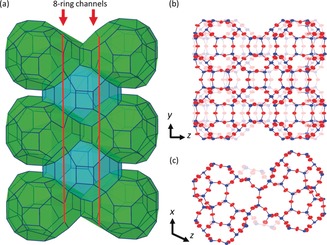
a) Schematic representation of the crystal structure of SSZ‐27 with the heart‐shaped cavities (50T) in green, and the 42T cavities in blue. Each 42T cavity connects four heart‐shaped cavities through 8‐ring windows, forming one‐dimensional channels along the *y*‐axis. Red lines indicate the direction of a pair of 8‐ring straight channels. The 8‐ring channels are linked through the 42T cavities. The heart‐shaped cavities can be viewed as large side pockets. Views of the framework structure along the b) *x*‐ and c) *z*‐axes showing the ADPs at 50 % probability. Red Si, blue O.

Preliminary characterizations of SSZ‐27 using XRPD revealed several unindexed peaks, which were identified as coming from quartz and SSZ‐26 (**CON**) impurity phases (Supporting Information). In view of the fact that obtaining a pure sample of SSZ‐27 was proving to be difficult (more on this below), we opted to collect electron diffraction (ED) data using the continuous rotation method,^11^, ^12^ which offers a combination of fast acquisition times and high data completeness, because all of reciprocal space is sampled. We used a recently developed crystal tracking routine to ensure that the crystal stayed in the electron beam during rotation^13^ implemented in the software Instamatic.^14^ This makes it easier to collect data with high rotation ranges, and at the same time enables more accurate integration of the reflection intensities for structure refinement. Diffraction data were collected on eighteen isolated crystals between 200 and 1000 nm in size over three sessions (Supporting information Table S2 and Figure S2). Unit cell determination, indexing, and intensity integration were performed using XDS^15^ (Supporting information Tables S4 and S5). The patterns of fourteen crystals could be indexed with very similar unit cells, with mean lattice parameters of *a*=24.12(14) Å, *b*=13.81(6) Å, *c*=25.07(10) Å, *β*=115.19(18)° in space group C2/m , matching the unit cell found for SSZ‐27 in our preliminary XRPD assessment (Supporting Information). The cell parameters of SSZ‐27 were optimized using the synchrotron XRPD data and the Pawley^16^ profile‐fitting routine in TOPAS 5,^17^ yielding a good profile fit with *a*=23.29 Å, *b*=13.37 Å, *c*=24.38 Å, *β*=114.23°. Several of the fourteen ED data sets could be used for structure determination using the programs FOCUS^18^, ^19^ and SHELXT,^20^ revealing the framework structure of SSZ‐27 (Figure 3 a). The remaining four crystals had a unit cell matching SSZ‐26 (**CON**). A detailed characterization of the SSZ‐26 crystals can be found in the Supporting Information.

**Figure 4:**
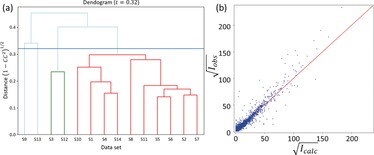
a) Dendrogram showing the correlation between the SSZ‐27 data sets (crystals S1–S14) corresponding to the data shown in Table S4 (Supporting Information). The cut distance set at 0.32 is represented by the horizontal line. Two clusters (green, red) can be identified. b) $\sqrt{I_{obs}}$ vs. $\sqrt{I_{calc}}$ plot for the refinement of SSZ‐27. The refinement details can be found in Table S7 (Supporting Information).

Because the crystal system of SSZ‐27 is monoclinic, none of the ED data sets had a completeness of more than 90 %, which is required for a good structure refinement. Therefore, to improve data completeness and redundancy, several data sets were merged. We performed hierarchical cluster analysis (HCA) on the fourteen data sets using the algorithm described by Giordano and co‐workers^21^ to find the optimal selection of data sets for merging. We have found this approach to be useful for dealing with large multi‐crystal ED data sets.^22^ The distance metric *t*, which defines the similarity between the data sets, is derived from the correlation coefficients of the common reflection intensities ($CC_{I}$ ) in pairs of data sets, and the “average” linkage method is used. The clusters can be visualized in a dendrogram (Figure 4 a), which facilitates finding a suitable cut distance, although finding the right value involved some trial and error. Finally, a cut distance *t*=0.32, equivalent to $CC_{I}=0.95$ , was used. In our experience with HCA, clusters with *t*<0.40 usually result in useful data sets. Herein, two clusters were obtained, where the largest cluster, consisting of 10 data sets (shown in red in Figure 4 a), comprised the highest data quality (assessed via $CC_{1/2}$ 23) and completeness (Supporting Information Table S6). The scripts for cluster analysis are available online from https://github.com/stefsmeets/edtools.

The ten data sets belonging to the largest cluster were merged using XSCALE^15^ and structure refinement was initiated using the lattice parameters obtained from the XRPD data, reasoning that these would be more accurate than those obtained from ED data. Indeed, this immediately resulted in more reasonable average Si−O bond lengths. Restraints on the Si−O distances and O‐Si‐O angles were not necessary. The atomic displacement parameters (ADPs) for all framework atoms were refined anisotropically. In the final stages of the refinement, the SWAT instruction was introduced to model the diffuse species in the channels (such as, the SDA, residual water, Na^+^). This instruction suppresses the contribution from the strong, low‐angle reflections where the contribution of extra‐framework species is strongest. This significantly reduced the *R*1 value, and resulted in more meaningful shapes for the ADPs (Figure 3 b,c). The refinement of the framework structure of SSZ‐27 converged with *R1*=0.178, *wR2*=0.486, and *S*=1.47 (Figure 4 b). Crystallographic details and the geometry of the zeolite framework are summarized in the Supporting information (Tables S6–S8).

Recently, we showed that physically meaningful anisotropic ADPs can be obtained from ED data, provided these are of sufficiently high quality.^13^ ADPs are known to act as a “fudge factor” for poor quality data. For this reason, they are often refined isotropically or their physical meaning is ignored. In the case of SSZ‐27, the anisotropic refinement of the ADPs was stable without the addition of restraints and their values are physically sensible (Figure 3 b,c). They are slightly elongated along the *z*‐direction, which we attribute to lower data redundancy along this direction. The ADPs of the O atoms are slightly larger than those for Si, and elongated in the direction perpendicular to the plane formed by the Si‐O‐Si bonds. The fact that reliable ADPs can be obtained for submicron‐sized crystals from ED data may open up new possibilities for studying the atomic vibrations or static disorder in zeolites that do not grow large enough for X‐ray single‐crystal analysis. Finally, we attempted to find the position of the SDA from the ED data. To do so, the SWAT instruction was removed, and a difference map generated. Although the difference map clearly revealed two large clouds of residual electrostatic potential (Supporting Information Figure S3), refinement of the SDA in the heart‐shaped cavity was not stable, so we had to conclude that the data do not support refinement of the SDA at this stage (see also the Supporting information).

### Framework Structure

**Figure 5:**
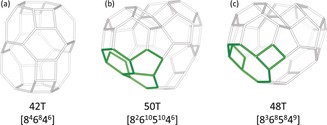
Schematic representation of a) the small 42T cavity, b) the heart‐shaped cavity observed in SSZ‐27, and c) the heart‐shaped cavity observed in ITQ‐55. The differences between the heart‐shaped cavities are highlighted in green.

**Figure 6:**
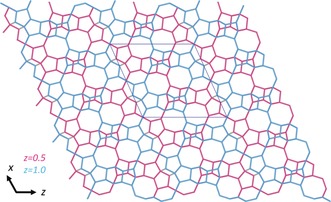
Schematic representation of the framework structure of SSZ‐27 along the *y*‐axis showing the arrangement of the heart‐shaped and 42T cavities in the *xz*‐plane. The structure can be described in terms of layers that stack along the *y*‐axis, where pink depicts the *y*=0.50±0.25 layer and blue the *y*=1.00±0.25 layer. The layers are connected via 8‐rings that connect each 42T cavity with 4 heart‐shaped cavities in the adjacent layers, forming 1D channels running parallel to the *y*‐axis. O atoms are omitted for clarity.

The structure analysis confirmed that SSZ‐27 is indeed a small pore zeolite. As mentioned earlier, the data also revealed the presence of a small, but noticeable amount of SSZ‐26. The framework structure of SSZ‐27 is characterized by two types of cavities (Figure 5 a,b). The larger, heart‐shaped cavity consists of 50 T‐atoms ([8^2^6^10^5^10^4^6^]), and has two 8‐ring windows with free dimensions of 3.6×5.5 Å (Supporting Information Figure S4), which are shared with a smaller cavity of 42 T‐atoms ([8^4^6^8^4^6^]). Each 42T cavity, delimited by a 14‐ring perpendicular to the *z*‐axis, connects four heart‐shaped cavities together. Figure 6 shows the orientation of the heart‐shaped cavities with respect to the 42T cavities and the layer they form in the *xz*‐plane. Each layer is shifted by (1/2 , 1/2 , 0) with respect to the adjacent one. SSZ‐27 has a one‐dimensional channel system. A pair of linked straight 8‐ring channels runs along the *y*‐axis, alternating between single 42T and two heart‐shaped cavities. The heart‐shaped cavities can be viewed as large side‐pockets. This means that despite this being a small‐pore zeolite, the internal volume is relatively large (Figure 3).

Another small‐pore zeolite with a slightly different heart‐shaped cavity was reported recently by Bereciartua and co‐workers.^24^ The framework structure of this zeolite, ITQ‐55, consists of heart‐shaped cavities exclusively. These 48 T‐atom cavities ([8^3^6^8^5^8^4^9^]) are interconnected via three shared 8‐ring windows forming a 2‐dimensional channel system. The difference between the ITQ‐55 and SSZ‐27 heart‐shaped cavities is that two 4‐rings and one 8‐ring in the ITQ‐55 cavity are replaced by two 6‐rings and two 5‐rings in SSZ‐27 (Figure 5 b, c). Furthermore, the 8‐ring openings in ITQ‐55 appear to be more restricted (free dimensions: 2.1×5.8 Å and 3.4×5.3 Å) than those in SSZ‐27 (3.6×5.5 Å; Supporting Information Figure S5).

To our knowledge, the **EEI** 25, 26 framework type is the only one besides that of SSZ‐27 with small pores, a one‐dimensional channel system, and large side pockets. In our previous experiments, SSZ‐45 (**EEI**) showed a surprisingly low N_2_ microporosity (0.056 cm^3^ g^−1^),^26^ which appeared to be limited by the fact that the connection between the cavities involves two 8‐rings (i.e. forms a short channel) and is relatively rigid. In contrast, the micropore volume of 0.14 cm^3^ g^−1^ found for SSZ‐27 is much more in line with what is expected for a zeolite (Supporting Information Figure S1). We suspect that the pore configuration consisting of only a single 8‐ring window connecting the large cavities allows for much more efficient diffusion.

### Molecular Modelling and the Location of the SDA

**Figure 7:**
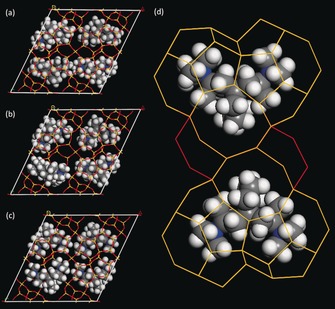
Modelling fit and energetics for a) isomer I (−3.66 kJ/Mol), b) isomer II (−3.31 kJ/Mol), and c) isomer III (−8.95 kJ/Mol) in the heart‐shaped cavities of SSZ‐27. d) close‐up view of isomer III. Grey C, white H, blue N.

The molecular modelling results show that the SDA is located exclusively in the heart‐shaped cavity, which fits around the SDA (isomer III) like a glove (Figure 7). At a maximum occupancy of 0.5 on a position of mirror symmetry, there is one SDA (with two possible orientations) per heart‐shaped cavity, or four (8 N^+^) per unit cell. Based on elemental analyses (Supporting Information Table S12), the Si:Al ratio of the framework was estimated to be 14.5, which translates to 7.5 Al per unit cell, so the charge from the SDA balances that of the framework nicely. This would indicate that there are no additional organic cations in the 42T cavity, where the second cloud of residual electrostatic potential was found, and that is consistent with the fact that the cavity is too small to accommodate an SDA cation. On the other hand, the elemental analysis also indicates the presence of a small amount of residual Na^+^ (approximately 1.0 per cell), which could be located in the 42T cavity with an occupancy of 0.5. This may indicate that the SDA is not fully occupied, which we have observed before.^27^, ^28^, ^29^ The rest of the 42T cavity is probably filled with residual water.

It has been observed experimentally,^30^ and predicted computationally^31^ that the SDA can direct the location of Al in aluminosilicates. We have previously observed this for borosilicates, in which B atoms are preferentially located in close proximity to the N^+^ centre in the SDA.^27^, ^31^ In SSZ‐27, the SDA appears to be located exclusively in the heart‐shaped cavity, which suggests that the Al is likely to be at the surface of this cavity too. We hypothesize that this means that all the active sites are accessible in the side pockets created by the heart‐shaped cavities, whereas the main straight channels are relatively free of active sites, and this might reduce the potential for blockage.

### NMR Spectroscopy

**Figure 8:**
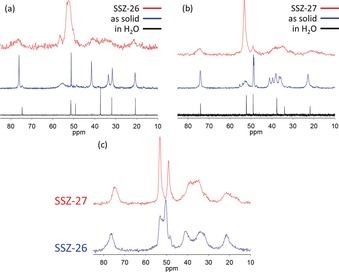
^13^C MAS NMR spectra for a) SSZ‐26 (red), isomer I in the solid state (blue) and in water (black), b) SSZ‐27 (red), isomer III in the solid state (blue) and in water (black). c) ^13^C CPMAS‐NMR (0.5 ms cross‐polarization contact time) spectra for SSZ‐26 and SSZ‐27.

Figure 8 shows the ^13^C MAS NMR spectra for the as‐made products in runs to make SSZ‐26 (with isomer I) and SSZ‐27 (with isomer III). Also shown are the spectra for the two separate isomers (I and III) in the crystalline solid state and in solution. There are clear differences in the ^13^C MAS NMR spectra of the two isomers, but in both cases the lines become broader and less distinctive when they are entrapped in the SSZ‐26 and SSZ‐27 framework structures. Note that the ^13^C resonances in the crystalline solid are more complicated than those in solution phase,^4^ especially for peaks in the 35–45 ppm region for isomer I and the 45–65 ppm region for isomer III. The change may be attributed to the generation of less symmetric crystalline stacking of molecules during solidification. Therefore, the ^13^C NMR spectra of the two zeolites do not provide a clear view of the structural difference between the isomers. However, ^13^C cross‐polarization MAS (CPMAS) spectra in Figure 8 c differentiate resonances of methylene carbons in the 5‐membered rings (30–45 ppm) as well as the methyl groups.

**Figure 9:**
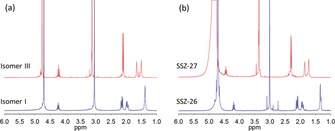
Liquid‐phase ^1^H NMR spectra for a) pure isomers I and III, and b) organic compounds extracted after dissolution of SSZ‐26 and SSZ‐27.

To unambiguously show which isomer was occluded inside the two frameworks we dissolved the crystalline samples of SSZ‐26 and SSZ‐27 with HF and extracted the organic material for ^1^H NMR analysis. Besides a slight shift because of the acidic condition after dissolution with HF, the ^1^H NMR spectra (Figure 9) clearly demonstrate that the isomers recovered from SSZ‐26 and SSZ‐27 can be identified as I and III, respectively. This proves that both samples contain only a single isomer, and that no interconversion occurred during zeolite synthesis and crystallization.

On the other hand, the ED structure analysis clearly showed that SSZ‐26 is a minor component in the SSZ‐27 sample studied. Additional modelling studies (Supporting information Table S13 and Figure S9) show that while isomer I significantly favours the formation of SSZ‐26 (−7.6 kJ mol^−1^ Si) over SSZ‐27 (−3.66 kJ mol^−1^ Si), isomer III only favors SSZ‐27 slightly (−8.95 kJ mol^−1^ Si) over SSZ‐26 (−8.18 kJ mol^−1^ Si). Then it is perhaps not surprising that some SSZ‐26 formed in the presence of isomer III, albeit more slowly than SSZ‐27. Of course, the chemical environment of isomer III in SSZ‐26 will be slightly different from that in SSZ‐27 and this will contribute to the breadth of the ^13^C NMR signals, making a definitive interpretation of the NMR spectra difficult. Additional NMR studies using nuclear Overhauser effect spectroscopy (NOESY) to better probe the details of the SDA within the zeolite are planned.

### Additional Synthesis Studies

In an attempt to confirm the indications of the energy calculations that isomer I favors the formation of SSZ‐26 and isomer III SSZ‐27 experimentally, we performed a set of synthesis experiments where the two isomers (I and III) were used individually with and without seeding (Table 1). It can be seen that in the case of isomer III, which shows the favourable space‐filling (Figure 7), SSZ‐27 crystallizes. The formation is even faster if there is seeding with SSZ‐27. In contrast, the other two experiments yield no SSZ‐27 and eventually form SSZ‐26 after a much longer run period (4–5 times longer). In the process of determining the crystallization requisites for reproducing the synthesis of SSZ‐27, we found that higher temperatures (170 °C) favoured SSZ‐27 over SSZ‐26. The latter is typically made at 160 °C. While the zeolite synthesis literature does describe some examples of crystallization of relatively open structures at higher temperatures, the general trend in crystallization is to produce open, metastable structures at short reaction times and lower temperatures and then zeolites with progressively higher framework density (sometimes through structure transformation^33^) at longer reaction times and higher temperatures. The void volumes in zeolite structures have an inverse relationship to framework density, and the framework structure of SSZ‐26, which crystallizes at the lower temperature, is in fact more open than that of SSZ‐27.

**Table 1:** Crystallization selectivity for the use of 2 SDA isomers.

Run	Isomer	Seeded?	Result
**1**	III	Yes	Good (SSZ‐27 formed)
**2**	III	No	Slower crystallization of SSZ‐27
**3**	I	Yes	No SSZ‐27, eventually SSZ‐26
**4**	I	No	No SSZ‐27, eventually SSZ‐26 (slower than in run **3**)

Returning to the theme advanced in the introduction, alicyclic derivatized ring structures provide good guest molecules for the formation of cage‐based zeolites under the right synthetic conditions. The zeolite SSZ‐13 (**CHA**) was first discovered with a derivative of the polycyclic hydrocarbon, adamantane.^34^ Similarly, a very large number of piperidine ring derivatives will make SSZ‐35 (**STF**).^35^ The underlying aim in the synthesis was to produce guest molecules that have axes long enough to extend along the channels of a zeolite. This is true for the *anti*,*anti* isomeric derivative (isomer I) and, in fact, SSZ‐26 is the only zeolite made with this SDA. However, once both trimethyl ammonium groups have *syn*,*syn* configurations (isomer III), it is as though the molecule is beginning to fold in on itself to produce more of a cage structure. This was well‐illustrated in our previous work to produce a triquaternary imidazolium derivative off a central benzene ring, where the imidazolium charged groups folded in on themselves to stabilize the sizable cage of the small pore zeolite **LTA**.^36^

## Conclusion

Synthesis highlights in this work are that subtle changes in the rigid organo‐cation (SDA) stereochemistry can lead to entirely different zeolite (host lattice) structures as the framework develops around the guest molecule. In turn, as we continue to increase our database of known zeolite structures, and understand the spatial orientation of the guest SDA molecules in the lattice, our ability to create new pairings improves. That is, we can predict what type of host environment can accommodate the guest and in which orientation. Molecular modelling of the type seen herein can be used effectively to check on the feasibility of the orientation of the SDA in a prospective lattice. In addition, our understanding of how the inorganic conditions of a zeolite synthesis bias the potential for specific sub‐units in the developing structure provide another parameter in model building for creating space for a guest. The resurgence of discoveries related to ABC‐6 zeolites, small‐pore products with larger cages that host the organo‐cations, are a good example. The SSZ‐27 zeolite, rich in 5‐ and 6‐ring subunits (which typically requires a higher Si/Al synthesis regime) is found to have a good spatial consistency with one particular SDA isomer, and then yields a highly unusual framework structure.

At the same time, developments in electron diffraction methodology, both hardware and software, have reached a point where high‐quality data can be collected routinely on a large number of crystals. As the XRPD data were insufficient for structure refinement, ED data were collected on eighteen crystals, four of which belonged to an SSZ‐26 impurity phase, corroborating our conclusions from the molecular modelling studies that SSZ‐26 forms equally well using all three isomers. The other fourteen crystals belonged to the SSZ‐27 phase. Key to this study is the application of hierarchical cluster analysis that leads to an optimal selection of data sets for structure refinement. This, in turn, helps to obtain more reliable and precise atomic coordinates of the framework, but also to get a handle on the atomic displacements of the atoms. The fact that physically meaningful atomic displacement parameters can be obtained from submicron‐sized crystals opens up new possibilities for studying atomic motion (e.g. internal vibrations) and disorder (static or dynamic). The framework structure that emerged for SSZ‐27 is that of a small‐pore zeolite with a one‐dimensional channel system connecting heart‐shaped cavities with smaller cavities through 8‐rings. Through molecular modelling, we were able to establish that the SDA is located exclusively in the heart‐shaped cavity. It is assumed that the other cavity contains water.

## Conflict of interest

The authors declare no conflict of interest.

## Supporting information

As a service to our authors and readers, this journal provides supporting information supplied by the authors. Such materials are peer reviewed and may be re‐organized for online delivery, but are not copy‐edited or typeset. Technical support issues arising from supporting information (other than missing files) should be addressed to the authors.

Supplementary
