# Supplementary material for: SSZ‐27: A Small‐Pore Zeolite with Large Heart‐Shaped Cavities Determined by Using Multi‐crystal Electron Diffraction
Source: Angew Chem Int Ed Engl. 2019 Aug 21;58(37):13080–6. doi: 10.1002/anie.201905049 (PMC6773097; doi:10.1002/anie.201905049)
Supplement: Supplementary file 1 — Supplementary [file ANIE-58-13080-s001.pdf]

Angewandte Chemie
Eine Zeitschrift der Gesellschaft Deutscher Chemiker

Supporting Information

SSZ-27: A Small-Pore Zeolite with Large Heart-Shaped Cavities Determined by using Multi-crystal Electron Diffraction

Stef Smeets, Stacey I. Zones,* Dan Xie, Lukáš Palatinus, Jesus Pascual, Son-Jong Hwang, Joel E. Schmidt, and Lynne B. McCusker*

anie_201905049_sm_miscellaneous_information.pdf

Experimental Section

Preparation of [4.3.3.0] propellane-8,11-dione

The [4.3.3.0] propellane-8,11-dione is prepared as described previously.[1] 110 grams of 1,2 cyclohexane dione is reacted with 365 grams of dimethyl-1,3-acetone dicarboxylate in 750 mL of a buffer solution made from 4.8 grams of disodium phosphate, 0.9 grams of citric acid in 200 mL water and then 550 mL methanol. The target for the water portion is pH = 6.8. The reaction is stirred for several days. The tetra ester product is collected and hydrolyzed to produce the propellane dione. The tetra ester was refluxed in 300 mL of 6M HCL over a period of 7 hours. The solid product, upon cooling, can be checked by IR for the removal of all ester functionality.

Synthesis of the SDA.

The initial mixture of tertiary amine isomers (**Error! Reference source not found.**) is obtained by using the Leuckart reaction[2] to convert the propellane dione to the dimethylamine terminus and then reacting the diamine with methyl iodide. 33 g of the propellane dione was mixed with 33 g of dimethylformamide and 27 g of formic acid (88%) and then heated in a closed system at 180°C for 16 hours. The product is collected and dissolved in 100 mL of water. The pH of the solution is raised to above 13 by addition of 50% NaOH solution. Then, 5 extractions are carried out with 100 mL diethyl ether each time to recover the free amines from the aqueous phase. The extracts are dried over sodium sulfate overnight, combined and stripped down to leave an oil of the mixed amine isomers. The mixture of amines is dissolved in 100 mL chloroform and 21 g of methyl iodide (a molar excess) are added dropwise with the solution in an ice bath. Solids will precipitate out as the diquaternary ammonium product forms. The product can be collected after 2 days of stirring at room temperature by vacuum filtration and washing with diethyl ether. Isomers I or III can be collected by separate recrystallization approaches. For isomer I, 1.2 g of the mixed diquaternary ammonium salts were heated up in 10 mL of 100% ethanol (78°C) until some of the material has dissolved. Then water is added dropwise while at that temperature. At the point where the solution becomes clear, 0.60 mL of water is added. Cooling to room temperature in a freezer will eventually produce a crystalline product. A similar recrystallization but using acetonitrile will produce isomer III instead. 1 g of the reaction mixture is mixed with 13 mL acetonitrile and brought to reflux. Then 1 mL water is added to eventually produce a clear solution. Crystals of isomer III are obtained upon cooling in the freezer. The NMR spectra of isomer I and III are shown in **Error! Reference source not found.** (and also in ref [3] where the crystal structures are given).

Zeolite synthesis.

1 mmole of the SDA (Isomer III) in the OH form, in 2.5 g of water, was added into a Teflon liner for a 23 mL Parr reactor. Next, 2 g of 1 N NaOH solution was added, followed by 1 g of water, and NaY Zeolite (CBV 100, Zeolyst International, SiO/Al_2O mole ratio=5.1) as the aluminum source. Finally, 0.60 g of CAB-O-SIL(R) M5 fumed silica (Cabot Corporation) was added. The liner was capped and placed within a Parr steel autoclave reactor. The autoclave was then fixed in a rotating Spit (43 rpm) within an oven and heated at 170°C for 7-10 days. The solid products were recovered, washed thoroughly with deionized water and dried.[4]

Scanning electron microscopy.

Scanning electron micrographs (**Error! Reference source not found.**) were obtained using a JEOL JSM 6700F instrument.

Micropore.

The porosity features of the zeolite were characterized with nitrogen adsorption at 77 K on a Micromeritics Tristar 3030 apparatus. The sample was degassed at 400°C in nitrogen flow overnight before adsorption analysis. The specific surface area was calculated according to the Brunauer-Emmett-Teller (BET) model in p/p_0 = 0.05-0.2 pressure range. Micropore volume was obtained by the *t*-plot method, using adsorption data collected in p/p_0 = 0.015-0.4 pressure range. The total pore volume of the sample is measured at p/p_0 = 0.99 pressure point. The micropore volume of SSZ-27 is 0.136 cm^3/g, with a total pore volume of 0.229 cm^3/g, and a surface area of 347 m^2/g.

X-ray powder diffraction.

Lab X-ray powder diffraction (XRPD) data were collected on a PANAlytical X'pert Pro MRD diffractometer with Cu Kα radiation (wavelength = 1.5418 Å; **Error! Reference source not found.**). Synchrotron X-ray powder diffraction (XRPD) data were collected on as-synthesized samples of SSZ-27 (non-seeded) in 0.3 mm capillaries on the Materials Science beamline at the Swiss Light Source in Villigen, Switzerland[5] using a wavelength of 0.70872 Å and a Mythen II detector.

Electron diffraction.

ED data suitable for structure refinement were collected using the continuous rotation method[6] on a JEOL JEM-2100 with a LaB_6 filament at 200 kV using the software *Instamatic*[7] following the crystal tracking procedure described in ref. [8]. Samples were prepared by crushing a powder of SSZ-27 (non-seeded), which was then dispersed in ethanol, and ultrasonicated for 1 to 5 minutes. A drop of the dispersion was deposited onto a 3 mm copper TEM grid with continuous carbon film (CF400-Cu-UL from Electron Microscopy Sciences) or lacey carbon film (Cu150P from Okenshoji Co., Ltd), and the ethanol was allowed to evaporate. The raw ED data from all 18 crystals and processing files are available from https://dx.doi.org/10.5281/zenodo.2633052.

Molecular modeling.

To gain more knowledge as to which isomer might best promote the formation of SSZ-27, molecular modeling on the location and the van der Waals (vdW) interaction energy for each of the three SDA isomers with the SSZ-27 framework were carried out using the Materials Studio software.[9] The CVFF forcefield[10] was selected for the calculation and the most stable locations of the molecules were obtained by simulated annealing. The host framework was assumed to be all silica, and the unit cell was fixed during the calculation. The best fit was achieved with isomer III (**Error! Reference source not found.**), giving the interaction energy of -8.95 kJ/mol per T atom. The other two isomers provided a much poorer fit, the interaction energy was -3.66 kJ/mol per T atom for isomer I and -3.31 kJ/mol per T atom for isomer II. These results were then used to guide further syntheses of SSZ-27.

NMR.

^{13}C solid state NMR experiments were performed using a Bruker DSX-500 spectrometer and a 4 mm Bruker MAS probe. Solid powders of isomers I and III and zeolite powders SSZ-26 and SSZ-27 were each packed into 4 mm rotors and spun at 8 kHz to record the ^{13}C signal after a single pulse (direct polarization) or ^{1}H cross-polarization (CP). Typical rf power for the ^{13}C channel was 62.5 kHz while that for the ^{1}H channel was adjusted to satisfy CP conditions under sample spinning or to perform high power decoupling. Isomers were dissolved into DDI water and ^{13}C spectra of the solutions were collected under MAS conditions. All ^{13}C spectra reported are referenced to TMS at 0 ppm. ^{1}H liquid phase NMR experiments were performed using a Bruker UltraShield™ 400 Plus and all spectra were recorded in D_2O. For

the HF dissolution experiments, 100 mg of as-made zeolite was suspended in water and then HF was added to dissolve the zeolite framework. **Caution: Take all appropriate safety measures when working with HF.** Then the HF was neutralized with an excess of 1N NaOH. The water was evaporated under a stream of nitrogen and then the SDA was extracted from the remaining solids using D_2O for NMR analysis.

Preliminary structure determination of SSZ-27

Initially, single-crystal electron diffraction (ED) data were collected on a Philips CM120 with LaB_6 filament at 120 kV using discrete rotation steps of 1° combined with precession electron diffraction with a precession angle of 1°. The discrete rotation precession ED data could be indexed using the software *JANA2006*[11] with a monoclinic lattice, *a*=23.952 Å, *b*=13.792 Å, *c*=24.958 Å, α=90.25°, *β*=115.71°, *γ*=90.15°. From the reflection conditions, the space group was deduced to be $C2/m$. Reflection intensities were extracted using the program *PETS.*[12] The framework structure was determined initially using the zeolite-specific program *FOCUS,*[13,14] and shortly thereafter also *ab initio* using the charge flipping algorithm[15] implemented in the program *Superflip*[16]. The framework structure matches the one that was eventually obtained from the continuous rotation ED data.

Structure refinement using XRPD data

Structure refinement using the synchrotron X-ray powder diffraction (XRPD) data was attempted using the program *TOPAS 5.*[17] The background was estimated and subtracted manually, and updated during the course of the refinement. Because the lattice parameters from electron diffraction (ED) data can be off by up to 1.0 Å, the lattice parameters of SSZ-27 were optimized using the Pawley[18] profile-fitting routine in *TOPAS*, yielding a good profile fit (R_{wp}=17.20) with *a*=23.98Å, *b*=13.32Å, *c*=24.61, *β*=114.35°, but with some impurity peaks. However, after the model for the framework structure was introduced, the calculated diffraction pattern did not match the observed data at all. After several attempts to fit the profile, we realized that the lattice parameters were trapped in a local minimum, partly caused by a strong impurity reflection at 11.9° that was later identified as being the 110/101 of a quartz phase, and several others from a suspected SSZ-26 (**CON**) phase. After a few attempts to adjust the lattice parameters manually, we obtained a new unit cell: *a*=23.29 Å, *b*=13.37 Å, *c*=24.38 Å, *β*=114.23° that produced a better match between the observed and calculated patterns (Figure S10). Unfortunately, the presence of impurities hindered structure refinement using the XRPD data, which did not converge. In view of the fact that obtaining a pure sample of SSZ-27 was proving to be difficult and that the previously collected ED data were not of sufficient quality to permit structure refinement, new ED data were collected. The hope was that these new data could give some insight into the nature of the impurities, and that isolated SSZ-27 crystals could be located to provide ED data of a quality that would allow a reliable structure refinement.

Locating the SDA from the ED data

In an attempt to find the position of the SDA in as-synthesized SSZ-27, the SWAT instruction was removed, and a difference map generated (Figure S5). The very clean difference map clearly revealed two large clouds of residual electrostatic potential in the two different cavities. The coordinates obtained from the molecular modeling were used as a starting position for the SDA. The SDA was refined as a rigid-body model (AFIX 6). Unfortunately, the position of the SDA was not stable, and it tended to drift towards the framework. Therefore, the SDA was weakly restrained to the mirror plane, as indicated by the modeling work. The occupancy and U_{iso} values were highly correlated, so the occupancy was fixed

at the expected value of 0.5, but the U_{iso} values did not converge and rose to physically meaningless values of well over 1.0. With more reasonable values of U_{iso} (*i.e.* ≤ 0.1), the $R1$ values were much larger than in the absence of the SDA. Several attempts with different combinations of data sets were made, but to no avail. Although the difference map clearly shows residual electrostatic potential in the cavities, we had to conclude that the data do not support refinement of the SDA at this stage.

SSZ-26 minor phase

From the ED data, four crystals had a related, but different *C*-centered unit cell from the one identified as the main phase (Table S3), with mean lattice parameters of *a*=23.40(13) Å, *b*=13.77(7) Å, *c*=12.73(7) Å, *β*=110.89(18)°. For each of these four data sets, intensities were extracted in the space group $C2/m$. Initially, structure determination with *FOCUS*[14] on a few of these data sets was performed, and each revealed a **CON**-type framework structure.[19] After applying hierarchical cluster analysis, three data sets were merged (Table S9) and these data were used for structure determination using *SHELXT*.[20] All framework atoms were revealed and correctly identified. The framework structure was then refined using *SHELXL*. Diffuse solvent was modelled using the SWAT command. All atoms were refined anisotropically, using rigid-bond restraints (RIGU) to maintain reasonable ADPs. The average Si—O distance refined to 1.643 Å. Therefore, the unit cell axes (*a*, *b*, *c*) were scaled down by 2.6% to bring the average Si—O distance to a more reasonable 1.60 Å. The resulting lattice parameters are also more consistent with those published for SSZ-26.[21] A difference map was generated, but no shape corresponding to the SDA could be discerned. Instead, it appears that the framework itself is disordered. The peaks in the difference map can be matched with a shift of the framework by ½ along *z*. This could be an indication of stacking faults, as has been observed previously for SSZ-26/SSZ-33.[22] This aspect of the structure has not been investigated further. The refinement of the framework structure of SSZ-26 converged with $R1$ = 0.255, $wR2$ = 0.5432, and S = 1.40 (Table S10, Figure S9). The geometry of the framework is summarized in Table S11.

Table S1. Representative pore and dimensionality types for synthesized zeolites.*

Pore type	Dimensionality	Example	Notes
Small	1D	**MTF**	
Small (9-ring)	1D	**STT**	Crossed by 7-rings
Small (9-ring)	2D	**PWO**	
Small	3D	**CHA**	
Small × Medium	2D	**FER**	
Small × Large	2D	**MOR**	
Small × Med. × Large	3D	**IWW**	
Medium	1D	**TON**	
Medium	2D	**MEL**	
Medium	3D	**MFI**	
Medium × Large	2D	**SFS**	
Medium × Large	3D	**CON**	
Large	1D	**AFI**	
Large	2D	**SSF**	
Large	3D	**FAU**	
Extra-large	1D	**UTD**	14-ring pore
Extra-large × Medium	2D	***CTH**	14 × 10-ring
Extra-large × Large	2D	**UTL**	14 × 12-ring

*The 3-letter framework-type codes given can be used to find the structure details in the Database of Zeolite Structures (www.iza-structure.org/databases). Some of the materials discovered with rings greater than 14-ring, e.g. 18-ring and so on, have not been included in this table as they have not been widely studied in terms of either catalysis or how the organic guest fits into these very large pores.

Table S2. ED data collection parameters for all crystals.

#	Rotation range per image (°)	Total rotation range (°)	Exposure time per image (s)	Total exposure (s)	Number of frames	Number of images	Spot size
S1*	0.2825	125.14	0.6	265.8	397	44	4
S2*	0.2829	119.94	0.6	254.4	380	42	4
S3	0.2828	122.16	0.6	259.2	387	43	4
S4*	0.2320	122.97	0.5	271.4	477	53	1
S5*	0.2320	139.18	0.5	307.2	540	60	1
S6*	0.2321	135.29	0.5	298.4	525	58	1
S7*	0.2349	125.19	0.5	276.4	480	53	3
S8*	0.2366	100.79	0.5	220.9	384	42	4
S9	0.2335	102.51	0.5	224.7	395	43	3
S10*	0.2345	101.09	0.5	223.6	388	43	3
S11*	0.2373	117.46	0.5	256.7	446	49	3
S12	0.2348	82.90	0.5	183.1	318	35	3
S13	0.2330	103.92	0.5	231.3	402	44	3
S14*	0.2351	122.71	0.5	270.8	470	52	2
C1*	0.2332	126.65	0.5	271.5	487	54	2
C2*	0.2812	131.30	0.6	280.2	419	47	3
C3*	0.2353	97.65	0.5	215.3	374	41	3
C4	0.2350	125.75	0.5	277.5	482	53	3

*Used for structure refinement

Table S3. Summary of the ED data indexing results.

#	Space group	***a*** **(Å)**	***b*** **(Å)**	***c*** **(Å)**	***α*** **(°)**	***β*** **(°)**	***γ*** **(°)**	Volume (Å^3)	Phase
S1*	$C2/m$	23.98	13.81	24.96	90	115.22	90	7478.5	SSZ-27
S2*	$C2/m$	24.26	13.83	25.07	90	115.34	90	7599.8	SSZ-27
S3	$C2/m$	23.97	13.86	25.04	90	115.02	90	7539.3	SSZ-27
S4*	$C2/m$	24.24	13.98	25.16	90	114.94	90	7727.4	SSZ-27
S5*	$C2/m$	24.30	13.89	25.19	90	115.64	90	7664.4	SSZ-27
S6*	$C2/m$	24.07	13.84	25.13	90	115.38	90	7564.4	SSZ-27
S7*	$C2/m$	24.09	13.74	25.06	90	114.84	90	7529.7	SSZ-27
S8*	$C2/m$	24.14	13.82	24.90	90	115.10	90	7522.0	SSZ-27
S9	$C2/m$	23.94	13.76	24.94	90	114.93	90	7448.3	SSZ-27
S10*	$C2/m$	23.93	13.74	24.92	90	115.33	90	7400.7	SSZ-27
S11*	$C2/m$	24.18	13.80	24.94	90	115.28	90	7521.9	SSZ-27
S12	$C2/m$	24.15	13.77	25.09	90	115.44	90	7534.3	SSZ-27
S13	$C2/m$	24.66	13.76	25.07	90	115.38	90	7683.1	SSZ-27
S14*	$C2/m$	24.01	13.82	24.94	90	115.37	90	7476.1	SSZ-27
C1*	$C2/m$	23.23	13.69	12.69	90	111.09	90	3766.5	SSZ-26 (**CON**)
C2*	$C2/m$	23.44	13.86	12.86	90	110.96	90	3900.3	SSZ-26 (**CON**)
C3*	$C2/m$	23.31	13.86	12.70	90	110.87	90	3833.9	SSZ-26 (**CON**)
C4	$C2/m$	23.60	13.74	12.70	90	110.50	90	3857.7	SSZ-26 (**CON**)

*Used for structure refinement

Table S4. Data quality indicators for the ED data collected on SSZ-27.

#	d_{max} (Å)	d_{min} (Å)	Total	Unique	Compl. (%)	$I/\sigma(I)$	R_{meas}	$CC_{1/2}$	$I/\sigma(I)^{asym.}$	$B_{overall}$
**S1***	11.7	0.8	11377	4679	58.4	3.44	12.1	99.5	9.44	7.82
	11.7	1.0	8523	2894	69.9	4.94	11.4	99.5		
	1.0	0.8	2854	1785	46.1	1.01	67.3	84.2		
**S2***	12.1	0.8	10418	5496	67.5	1.83	17.1	99.6	9.13	6.87
	12.1	1.0	7375	3160	75.4	2.80	16.1	99.6		
	1.0	0.8	3043	2336	59.2	0.52	95.0	80.5		
**S3**	11.7	0.8	11095	5789	71.8	1.85	23.0	97.7	5.69	5.73
	11.7	1.0	7839	3321	79.7	2.65	21.8	97.7		
	1.0	0.8	3256	2468	63.3	0.78	79.5	78.1		
**S4***	11.8	0.8	15862	5261	63.6	1.93	19.2	99.1	6.44	8.68
	11.8	1.0	8274	2693	63.3	3.13	17.0	99.1		
	1.0	0.8	7588	2568	63.9	0.67	121.6	75.4		
**S5***	12.1	0.8	19791	7348	89.5	2.35	18.0	97.2	7.64	8.13
	12.1	1.0	10213	3800	89.8	3.66	15.7	97.2		
	1.0	0.8	9578	3548	89.2	0.95	94.7	86.8		
**S6***	12.0	0.8	20161	6988	86.4	1.00	31.6	96.4	6.03	9.19
	12.0	1.0	10561	3629	86.8	1.73	24.5	96.4		
	1.0	0.8	9600	3359	85.9	0.20	379.7	24.3		
**S7***	12.0	0.8	12128	4784	59.3	3.00	15.3	98.7	7.07	7.04
	12.0	1.0	9149	3022	72.6	4.05	14.6	98.7		
	1.0	0.8	2979	1762	45.1	1.20	53.5	91.6		
**S8***	12.0	0.8	9607	4306	53.5	3.86	13.4	98.5	7.67	6.45
	12.0	1.0	7261	2802	67.4	4.92	13.0	98.5		
	1.0	0.8	2346	1504	38.6	1.88	33.7	93.8		
**S9**	11.6	0.8	8052	4939	61.9	1.71	20.3	98.0	5.43	7.24
	11.6	1.0	6194	3326	80.7	2.21	19.8	98.0		
	1.0	0.8	1858	1613	41.8	0.68	94.9	66.8		
**S10***	11.6	0.8	9178	4277	54.0	1.36	24.7	98.5	5.78	8.25
	11.6	1.0	7111	2797	68.1	1.93	23.5	98.5		
	1.0	0.8	2067	1480	38.8	0.30	187.1	34.8		
**S11***	12.1	0.8	11145	4988	61.9	5.16	8.6	99.7	11.48	7.08
	12.1	1.0	7618	2984	71.8	7.09	8.1	99.7		
	1.0	0.8	3527	2004	51.4	2.28	30.1	97.2		
**S12**	12.1	0.8	7935	3692	45.7	3.53	16.7	98.4	5.63	6.30
	12.1	1.0	5935	2347	56.4	4.39	16.3	98.4		
	1.0	0.8	2000	1345	34.4	2.03	32.9	91.5		
**S13**	11.7	0.8	9855	4778	58.1	2.39	17.8	99.3	5.61	6.97
	11.7	1.0	7460	3047	71.7	3.20	17.2	99.3		
	1.0	0.8	2395	1731	43.5	0.98	56.8	84.7		
**S14***	12.0	0.8	11841	5359	66.9	2.59	21.6	95.8	4.47	7.21
	12.0	1.0	8955	3386	81.7	3.32	21.3	95.8		
	1.0	0.8	2886	1973	51.0	1.33	36.6	91.6		

*Used for structure refinement

Table S5. Data quality indicators for the ED data collected on the SSZ-26 minor phase.

#	d_{max} (Å)	d_{min} (Å)	Total	Unique	Compl. (%)	$I/\sigma(I)$	R_{meas}	$CC_{1/2}$	$I/\sigma(I)^{asym.}$	$B_{overall}$
**C1***	11.8	0.8	5776	2191	54.2	2.61	23.6	96.3	4.44	7.27
	11.8	1.0	4419	1468	70.0	3.29	22.9	96.2		
	1.0	0.8	1357	723	37.2	1.23	56.4	82.0		
**C2***	11.7	0.8	6115	3103	74.3	3.26	16.7	98.3	5.93	5.82
	11.7	1.0	4330	1784	82.5	4.59	16.2	98.3		
	1.0	0.8	1785	1319	65.6	1.45	43.8	84.3		
**C3***	9.3	0.8	3616	1940	47.3	2.07	26.0	94.2	3.68	6.56
	9.3	1.0	2741	1273	59.6	2.57	25.7	94.2		
	1.0	0.8	875	667	33.9	1.12	46.8	87.3		
**C4**	11.9	0.8	6536	3060	74.1	1.59	40.2	88.3	2.25	5.96
	11.9	1.0	4871	1791	83.4	2.08	40.0	88.3		
	1.0	0.8	1665	1269	64.0	0.90	52.0	69.4		

*Used for structure refinement

Table S6. Data scaling parameters for the merged data set of SSZ-27 (combining 10 crystals).

Shell	Full range	Low angle	High angle
Resolution range (Å)	11.59-0.80	11.59-1.0	1.0-0.80
Total data	130757	84285	46472
Unique Data	7914	4124	3790
Completeness (%)	98.8	99.6	97.8
$I/\sigma(I)$	4.53	7.01	1.82
R_{meas} (%)	31.5	29.3	141.3
$CC_{1/2}$	98.8	98.8	81.1
Overall B factor (Å^2)	8.17	-	-

Table S7. Crystallographic details for SSZ-27.

**Crystal data**	
Formula	[$Si_{116}O_{232}$]
Formula Weight (g/mol)	3485.22
Crystal System	monoclinic
Space group	$C2/m$ (No. 12)
a, b, c (Å)	23.2862(6), 13.3710(5), 24.3827(6)
α, β, γ (°)	90, 114.2248(19), 90
V (Å^3)	6923.3(4)
ρ (g/cm^3)	1.672
Framework density (Si/1000 Å^3)	16.8
F(000)	3480
**Data collection**	
Temperature (K)	293
Radiation (Å)	electrons, 0.02508
Number of merged data sets	10
d_{min}, d_{max} (Å)	0.77, 11.59
Dataset (h, k, l)	-29→29, -17→17, -31→31
Tot., Uniq. data, R_{int}	130755, 7913, 0.287
Observed Data [$I > 2.0\sigma(I)$]	4129
**Refinement**	
$N_{reflections}$, $N_{parameters}$, $N_{restraints}$	7913, 415, 0
$R1$ [$F^2 > 2.0\sigma(F^2)$], $wR2$, S	0.1782, 0.4855, 1.47
$(\Delta/\sigma)_{max}$	< 0.01
ρ_{min}, ρ_{max} (V/Å^3)	-0.27, 0.39

$w = 1/[\sigma^2 F_o^2 + (0.2P^2)]$ where $P = \frac{1}{3}(F_o^2 + 2F_c^2)$

Table S8. Selected bond angles and distances for SSZ-27.

	Si—O (Å)	O—Si—O (°)	Si—O—Si (°)
**Min.**	1.559	104.5	139.0
**Max.**	1.651	114.7	174.1
**Average**	1.60(2)	109.4(18)	152(9)
**Conventional**	1.61(1)	109.5(8)	145(10)

Table S9. Data scaling parameters for the merged data set (combining C1, C2, C3) for SSZ-26.

Shell	Full range	Low angle	High angle
Resolution range (Å)	11.59—0.80	11.59—1.0	1.0—0.80
Total data	15432	11353	4079
Unique data	3628	2058	1570
Completeness (%)	89.8	98.1	80.8
$I/\sigma(I)$	2.61	3.54	1.38
R_{meas} (%)	40.0	39.5	59.3
$CC_{1/2}$	91.9	91.8	77.9
Overall B factor from Wilson plot (Å^2)	6.03	-	-

Table S10. Crystallographic details for the SSZ-26 minor phase.

**Crystal data**	
Formula	$[Si_{56}O_{112}]$
Formula Weight (g/mol)	3365.04
Crystal System	monoclinic
Space group	$C2/m$ (No. 12)
a, b, c (Å)	22.783, 13.429, 12.401
α, β, γ (°)	90, 110.85, 90
V (Å^3)	3545.67
ρ (g/cm^3)	1.576
Framework density (Si/1000 Å^3)	15.8
F(000)	1680
**Data collection**	
Temperature (K)	293
Radiation (Å)	electrons, 0.02508
Number of merged data sets	3
d_{min}, d_{max} (Å)	0.78, 11.59
Dataset (h, k, l)	-27→27, -16→15, -14→14
Tot., Uniq. data, R_{int}	15342, 3628, 0.337
Observed Data $[I > 2.0\sigma(I)]$	1947
**Refinement**	
$N_{reflections}$, $N_{parameters}$, $N_{restraints}$	3628, 200, 171
$R1$ $[F^2 > 2.0\sigma(F^2)]$, $wR2$, S	0.2548, 0.5432, 1.40
$(\Delta/\sigma)_{max}$	< 0.01
ρ_{min}, ρ_{max} (V/Å^3)	-0.21, 0.41

$w = 1/[\sigma^2 F_o^2 + (0.2P^2)]$ where $P = \frac{1}{3}(F_o^2 + 2F_c^2)$

Table S11. Selected bond angles and distances for the SSZ-26 minor phase.

	Si—O (Å)	O—Si—O (°)	Si—O—Si (°)
**Min.**	1.564	105.6	135.9
**Max.**	1.646	113.7	162.5
**Average**	1.60(2)	109.4(18)	150(8)
**Conventional**	1.61(1)	109.5(8)	145(10)

Table S12. Elemental analysis on SSZ-27.

	element	wt%	Molar ratio	Expected
**CHN analysis**	C	13.17	18.00	18
	H	2.44	39.74	36
	N	1.63	1.91	2
**ICP-AES**	Al	2.27	1.00	
	Si	34.00	14.38	
	Na	0.266	0.14	

Table S13. Modeling energetics for isomers I and III in SSZ-26 and SSZ-27.

	Structure	SSZ-27 (kJ/mol Si)	SSZ-26 (kJ/mol Si)[1]
**Isomer I**	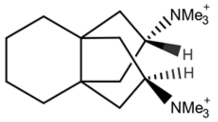	-3.66	-7.60
**Isomer II**	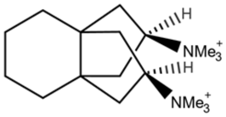	-3.31	-8.25
**Isomer III**	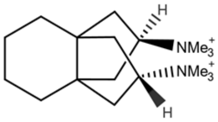	-8.95	-8.18

[1]Simulations for SSZ-26 were run with a reduced number of steps.

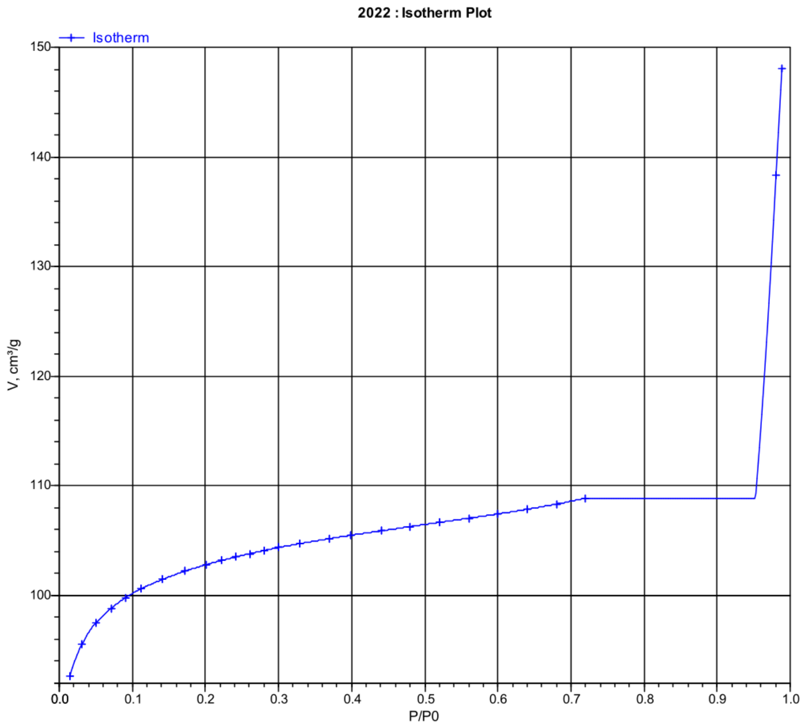

Figure S1. Isotherm plot for SSZ-27.

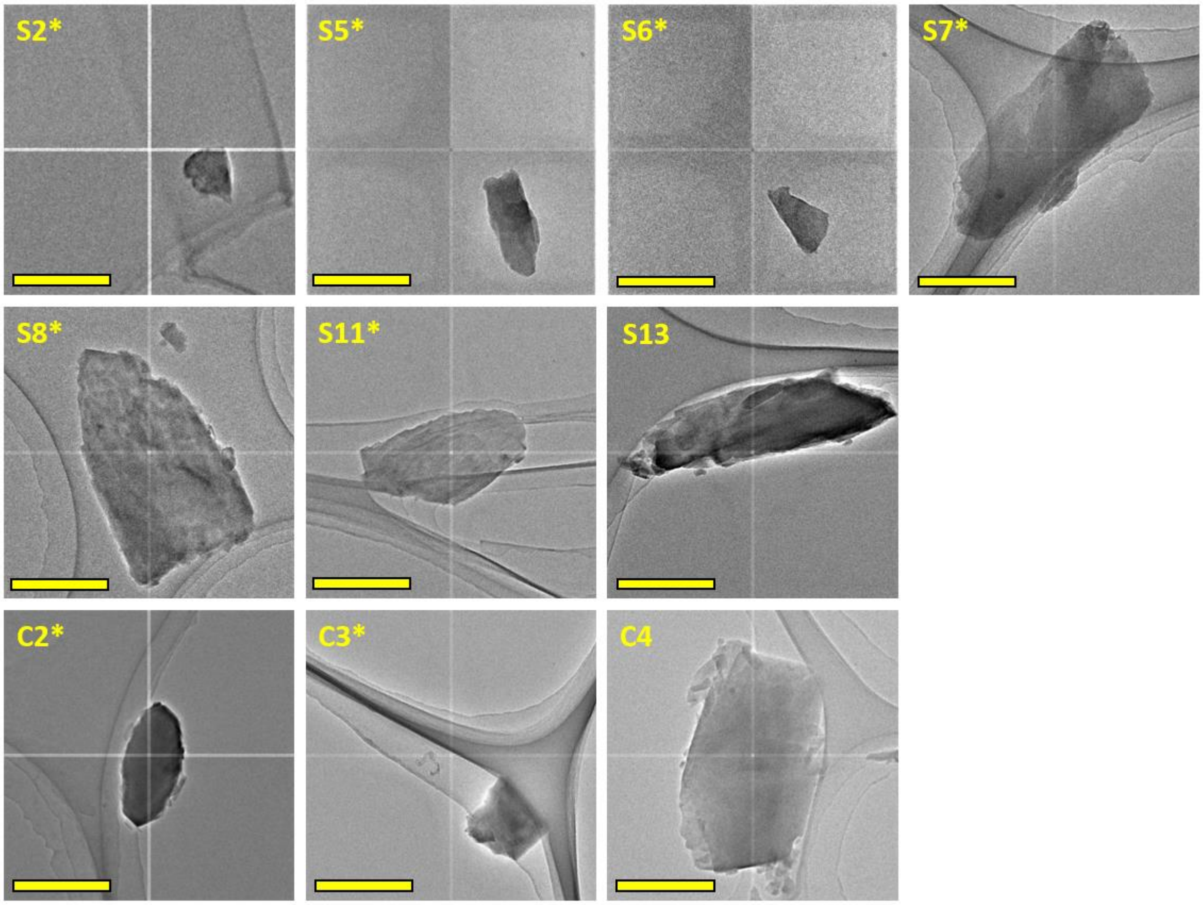

Figure S2. Selection of images from crystals from which ED data were collected. Those used for structure refinement are marked with a star (*). The scale bar corresponds to 400 nm.

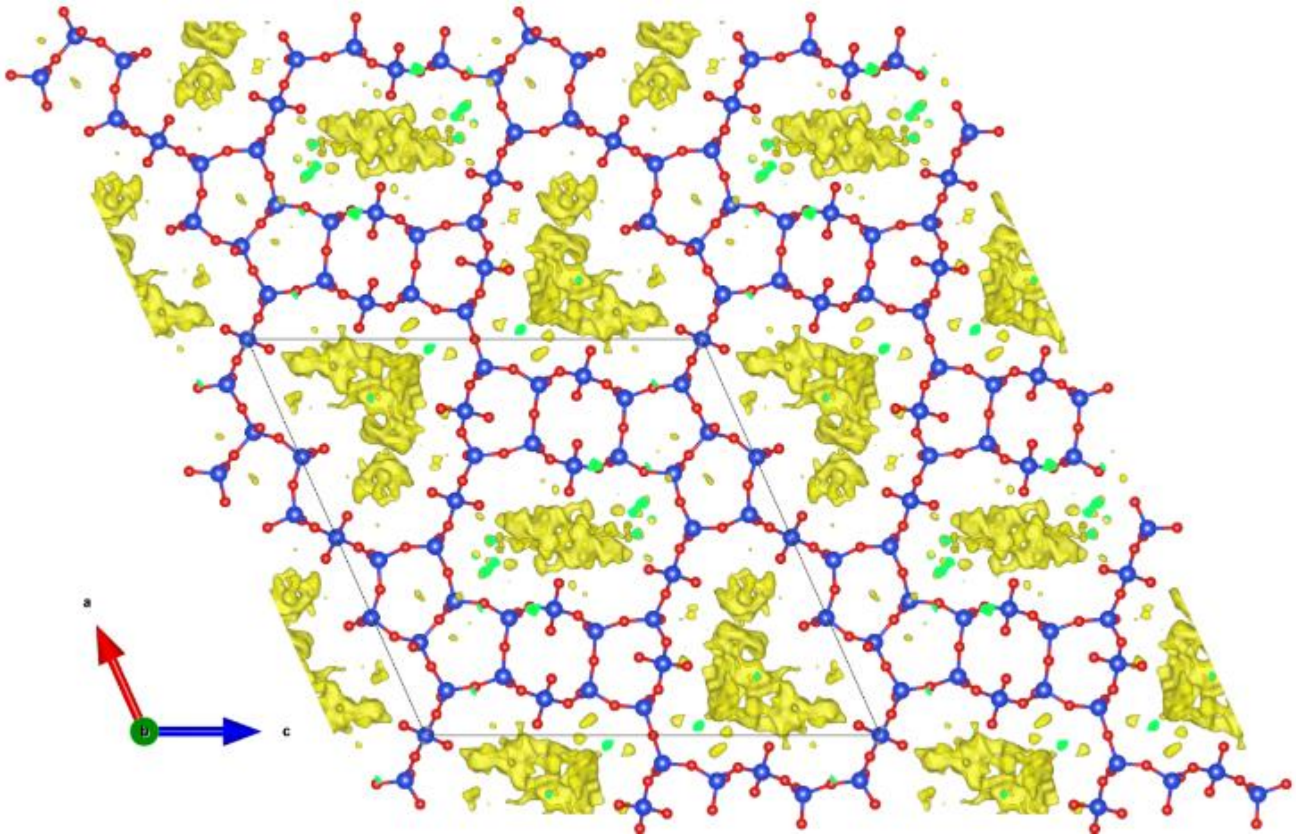

Figure S3. Difference map for SSZ-27 showing the residual electrostatic potential when only the framework atoms are present in the structural model. A cut from *z*=0.2 to *z*=0.8 is shown along the *y*-axis.

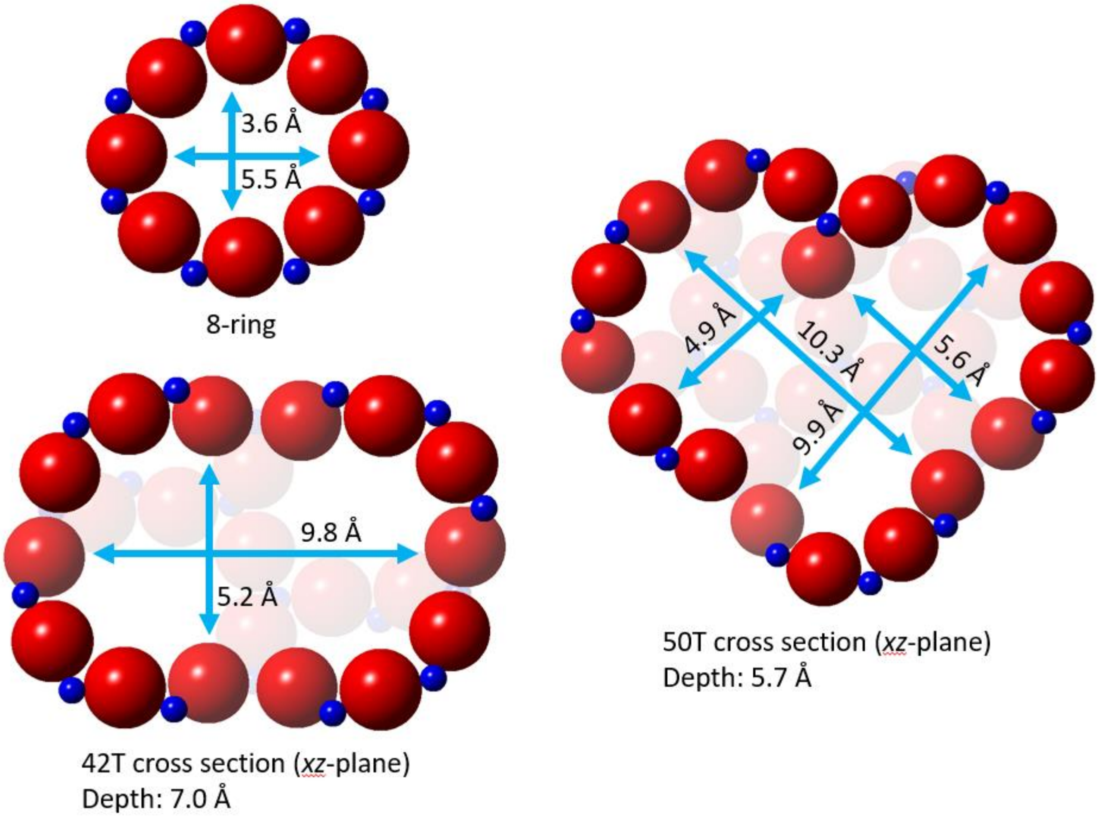

Figure S4. Effective pore opening of the 8-ring windows and the two types of cavities in SSZ-27. The van der Waals radius of O (1.35 Å) has been taken into account in calculating the distances.

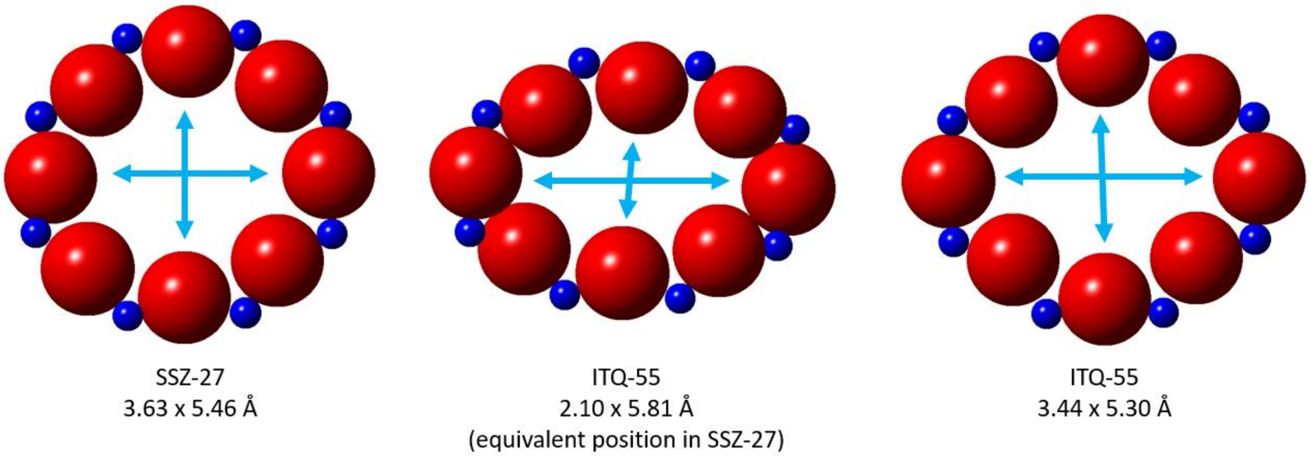

Figure S5. Effective pore opening of the 8-ring windows apparent in SSZ-27 and ITQ-55. The van der Waals radius of O (1.35 Å) has been taken into account in calculating the distances.

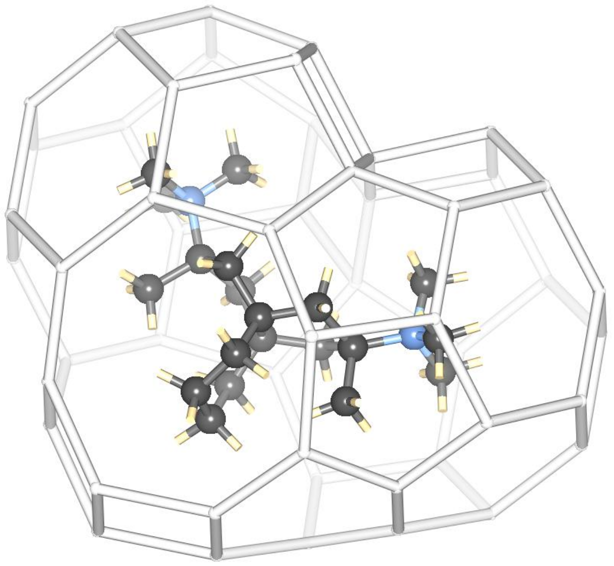

Figure S6. Schematic representation of the heart-shaped cavity showing the location of the SDA (Isomer III) obtained from molecular modelling.

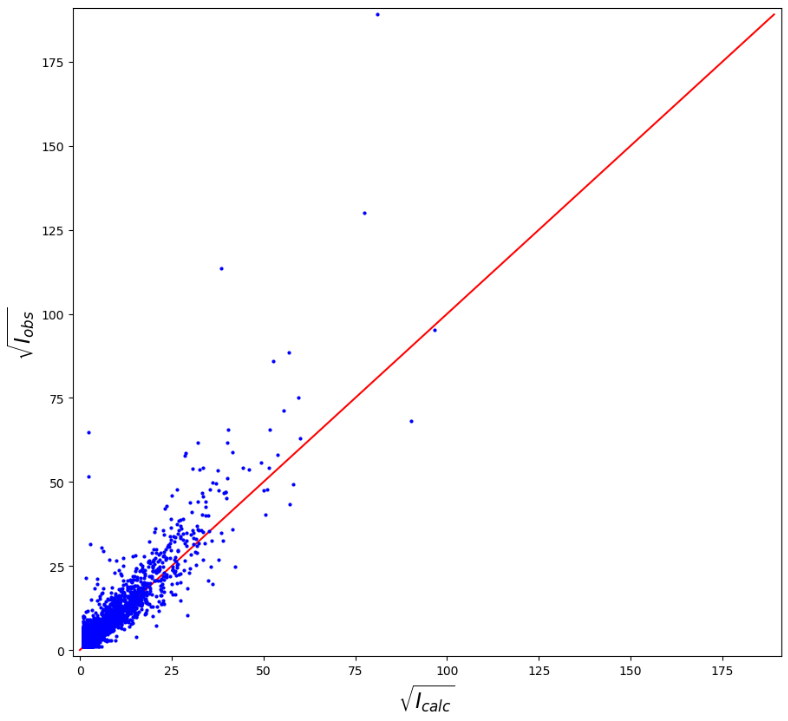

Figure S7. F_{obs} vs. F_{calc} plot for the refinement of the SSZ-26 minor phase. Generated using *ANAFCF* [23].

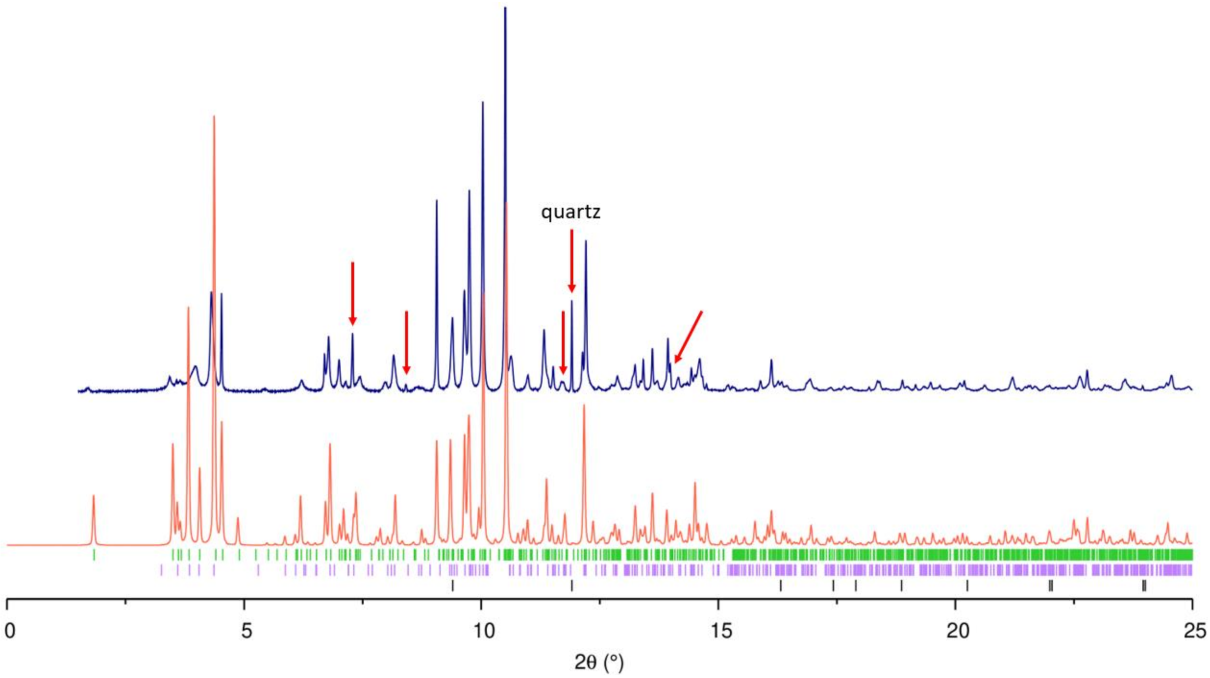

Figure S8. The observed (top, blue) and calculated (bottom, orange) diffraction patterns for as-synthesized SSZ-27. The calculated pattern corresponds to the final structure refined using ED data. The position of identified impurity peaks is indicated by red arrows. The coloured bars indicate the locations of the reflections corresponding to SSZ-27 (green), SSZ-26 (purple), and quartz (black).

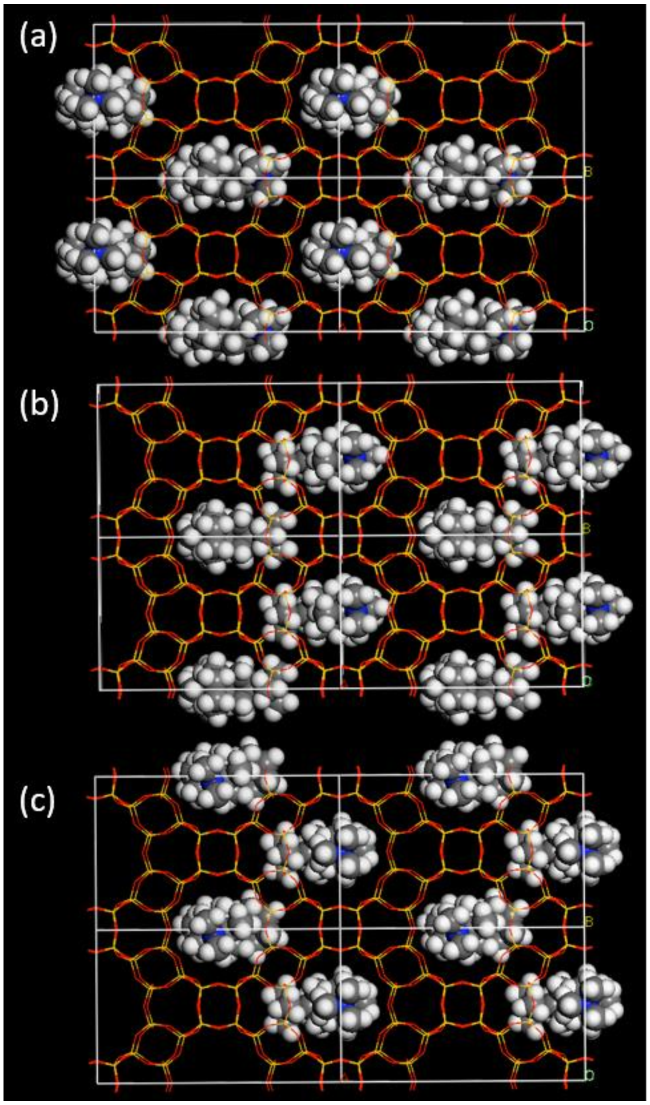

Figure S9. Modeling fit and energetics for (a) isomer I (-7.60 kJ/Mol), (b) isomer II (-8.25 kJ/Mol), and (c) isomer III (-8.18 kJ/Mol) in the channels of SSZ-26.

References

[1] S. Bhanumati, P. Ashkenazi, S. Migdal, D. Ginsburg, *Helv. Chim. Acta* **1983**, *66*, 2703–2706.
[2] R. D. Bach, *J. Org. Chem.* **1968**, *33*, 1647–1649.
[3] S. I. Zones, M. M. Olmstead, D. S. Santilli, *J. Am. Chem. Soc.* **1992**, *114*, 4195–4201.
[4] S. I. Zones, D. Xie, R. J. Saxton, *Method for Making Molecular Sieve SSZ-27*, **2017**, U.S. Patent 9,586,830 B2.
[5] A. Bergamaschi, A. Cervellino, R. Dinapoli, F. Gozzo, B. Henrich, I. Johnson, P. Kraft, A. Mozzanica, B. Schmitt, X. Shi, *J. Synchrotron Radiat.* **2010**, *17*, 653–668.
[6] I. Nederlof, E. van Genderen, Y.-W. Li, J. P. Abrahams, *Acta Cryst. D* **2013**, *69*, 1223–1230.
[7] S. Smeets, B. Wang, M. O. Cichocka, J. Ångström, W. Wan, *Instamatic 1.0*, Zenodo, Https://dx.Doi.Org/10.5281/Zenodo.1090388, **2018**.
[8] M. O. Cichocka, J. Ångström, B. Wang, X. Zou, S. Smeets, *J. Appl. Crystallogr.* **2018**, *51*, 1652–1661.
[9] *Materials Studio 6.0*, Accelrys, Inc., San Diego, **2012**.
[10] P. Dauber-Osguthorpe, V. A. Roberts, D. J. Osguthorpe, J. Wolff, M. Genest, A. T. Hagler, *Proteins: Struct., Funct., Bioinf.* **1988**, *4*, 31–47.
[11] V. Petříček, M. Dušek, L. Palatinus, *Z. Kristallogr. Cryst. Mater.* **2014**, *229*, 345–352.
[12] L. Palatinus, *PETS*, Prague: Institute of Physics of the AS CR, **2011**.
[13] R. W. Grosse-Kunstleve, L. B. McCusker, C. Baerlocher, *J. Appl. Crystallogr.* **1997**, *30*, 985–995.
[14] S. Smeets, L. B. McCusker, C. Baerlocher, E. Mugnaioli, U. Kolb, *J. Appl. Crystallogr.* **2013**, *46*, 1017–1023.
[15] G. Oszlányi, A. Sütő, *Acta Cryst. A* **2004**, *60*, 134–141.
[16] L. Palatinus, G. Chapuis, *J. Appl. Crystallogr.* **2007**, *40*, 786–790.
[17] A. A. Coelho, *J. Appl. Crystallogr.* **2018**, *51*, 210–218.
[18] G. S. Pawley, *J. Appl. Crystallogr.* **1981**, *14*, 357–361.
[19] C. Baerlocher, L. B. McCusker, D. H. Olson, *Atlas of Zeolite Framework Types*, Elsevier, **2007**.
[20] G. M. Sheldrick, *Acta Cryst. A* **2015**, *71*, 3–8.
[21] R. F. Lobo, M. E. Davis, *J. Am. Chem. Soc.* **1995**, *117*, 3766–3779.
[22] R. F. Lobo, M. Pan, I. Chan, R. C. Medrud, S. I. Zones, P. A. Crozier, M. E. Davis, *J. Phys. Chem.* **1994**, *98*, 12040–12052.
[23] M. Lutz, A. M. M. Schreurs, *LOGLOG and ANAFCF.*, Utrecht University, **2012**.
